# Platycodin D induces apoptosis through JNK1/AP-1/PUMA pathway in non-small cell lung cancer cells: A new mechanism for an old compound

**DOI:** 10.3389/fphar.2022.1045375

**Published:** 2022-11-22

**Authors:** Shuntai Chen, Qing Wang, Sarah Ming, Honggang Zheng, Baojin Hua, Hsin-Sheng Yang

**Affiliations:** ^1^ Department of Oncology, Guang’anmen Hospital, China Academy of Chinese Medical Sciences, Bejing, China; ^2^ Beijing University of Chinese Medicine, Bejing, China; ^3^ Department of Toxicology and Cancer Biology, College of Medicine, University of Kentucky, Lexington, KY, United States; ^4^ Markey Cancer Center, University of Kentucky, Lexington, KY, United States

**Keywords:** NSCLC, p53 upregulated modulator of apoptosis, mitochondral function, c-Jun N-terminal kinase, activator protein 1.

## Abstract

Platycodin D, a triterpenoid monomer, has been shown to possess an anti-tumor effect on various types of cancer. Although Platycodin D has been reported to suppress tumorigenesis, the detailed underlying mechanism remains elusive. Platycodin D treatment significantly reduced the cell viability, decreased the number of colonies, impaired the mitochondrial function, and induced apoptosis in non-small cell lung cancer (NSCLC) cells. To understand the mechanism by which platycodin D induces apoptosis, the expression levels of apoptosis-related proteins were examined, and we found that the expression of PUMA (p53 upregulated modulator of apoptosis) was upregulated upon platycodin D treatment. Knockdown of PUMA resulted in attenuation of platycodin D-induced apoptosis, indicating that PUMA up-regulation is essential for platycodin D to induce apoptosis. The induction of PUMA expression by platycodin D treatment was through activation of AP-1 since mutation of AP-1 binding site in the *PUMA* promoter abolished the *PUMA* promoter activity. In addition, the chromatin immunoprecipitation further demonstrated that platycodin D promoted AP-1 binding to PUMA promoter. Moreover, knockdown of JNK1, but not JNK2, significantly abolished the phosphorylation of c-Jun at Ser63 (a component of AP-1), decreased the platycodin D-induced expression of PUMA and cleaved caspase 3, indicating that platycodin D inhibits JNK1/AP-1 signaling pathway. Furthermore, immunohistochemical staining studies showed that tumors from the mice treated with platycodin D activated JNK by translocation of JNK into nuclei, increased phosphorylation of JNK and c-Jun at Ser63 in nuclei, and boosted the PUMA expression. Taken together, our *in vitro* and *in vivo* data revealed a novel mechanism by which platycodin D up-regulates PUMA to induce apoptosis through JNK1/AP-1 axis in NSCLC.

## 1 Introduction

Lung cancer is the leading cause of cancer-related deaths worldwide. The high mortality rate is not only due to a lack of effective early detection but also due to poor response to currently available therapies, which underscores the urgent need to decipher novel strategies for lung cancer intervention. Chinese herb *Platycodon grandiflorum*, containing many natural compounds with different configurations, is one of the medicines commonly used in traditional Chinese medicine to treat respiratory diseases (Deng et al., 2020). Platycodin D is a triterpenoid monomer isolated from *Platycodon grandiflorum*, which exhibits antioxidant, anti-obesity, and anti-inflammatory activities (Khan et al., 2016). Studies also found that cells treated with Platycodin D induce apoptosis on various types of cells including breast cancer cells (Yu and Kim, 2010), gastric cancer cells (Chun et al., 2013), prostate cancer cells (Zhou et al., 2015), bladder cancer cells (Zhang et al., 2020), and hepatic stellate cells (Liu et al., 2020). Although induction of apoptosis by platycodin D has been known for many years, the molecular mechanism is still not fully understood.

The B cell lymphoma 2 (Bcl-2) related proteins comprised three subgroups, i.e., Bcl-2 family, Bcl-2 associated X protein (Bax) family, and Bcl-2 homology 3 (BH3) only family (Campbell and Tait, 2018), regulates cellular apoptosis by controlling the release of cytochrome c from mitochondria to cytosol. Cytochrome c then binds with apoptotic protease-activating factor-1 to activate caspase cascades (Green, 2018). The Bcl-2 family proteins function as pro-survival proteins, while the Bax proteins and BH3 only proteins exhibit pro-apoptotic function. The p53 up-regulated modulator of apoptosis (PUMA) belongs to the BH3 family, which causes permeabilization of the mitochondrial outer membrane to release cytochrome c by either inhibiting pro-survival activity of the Bcl-2 family or directly activating Bax (Kim et al., 2006; Neise et al., 2008). PUMA was initially discovered to be up-regulated at the transcriptional level upon p53 binding to the p53-response elements on the *PUMA* promoter (Nakano and Vousden, 2001; Yu et al., 2001). In addition, transcription factors such as FOXO1, FOXO3a, CREB, Sp1, and Slug have been reported to up-regulate or down-regulate PUMA expression in response to various cellular stresses or stimulations (Li, 2021). For example, growth factor deprivation-induced apoptosis up-regulates PUMA expression *via* FOXO3a (Schubert et al., 2018). Thus, PUMA is a critical factor for maintaining the balance between apoptosis and survival under various physiological and pathological conditions. To date, the role of PUMA in platycodin D-induced apoptosis remains unknown.

In addition to Bcl-2 family proteins, Jun N-terminal kinase (JNK) also plays a crucial role in the regulation of apoptosis in response to cellular stress (Dhanasekaran and Reddy, 2008). JNK consists of three isoforms, i.e., JNK1, JNK2, and JNK3, which transactivates c-Jun by phosphorylation of Ser at positions 63 and 73 resulting in the formation of AP-1 transcription factor complex. The AP-1 transcription factor consists of a Jun (c-Jun, JunB, JunD)-Jun homodimer or Jun-Fos (c-Fos, FosB, Fra-1, Fra-2) heterodimer, which regulates a wide variety of gene expressions including pro-apoptotic and pro-survival genes (Dhanasekaran and Reddy, 2017).

In this study, we aimed to elucidate the molecular mechanism by which platycodin D induces apoptosis using non-small cell lung cancer (NSCLC) cells as a model system. We found that PUMA plays a critical role in the platycodin D-induced apoptosis and stimulation of PUMA expression by platycodin D through activation of the JNK1/AP-1 pathway. These findings in cultured cells were further validated in the tumors from the mice treated with platycodin D. Thus, we are the first to report that platycodin D induces cell death through JNK1/AP-1/PUMA axis, providing new mechanistic insights into platycodin D-induced apoptosis.

## 2 Materials and methods

### 2.1 Reagents

Platycodin D was purchased from the National Standard Network (China) or MedChemExpress (United State). SP600125 was purchased from BioVision. Both Platycodin D and SP600125 were dissolved in DMSO at a proper concentration and stored at −20°C.

### 2.2 Cell lines and culture

H1299, H2030, and A549 cells were purchased from the ATCC. H1299 and H2030 cells were cultured in RPMI-1640 medium and A549 cells were cultured in high glucose DMEM medium*.* All medium was supplemented with 10% FBS, 2 mmol/L L-glutamine, and 100 U/mL penicillin–streptomycin. Cells were cultured at 37°C in a humidified atmosphere of 5% CO_2_ in air.

### 2.3 Cell proliferation and clonogenic assays

Cell viability and proliferation was measured using CellTiter-Glo® Luminescent Cell Viability Assay kit (Promega) according to manufacturer’s protocol. Briefly, 2,000–3,000 cells were seeded onto each well of a 96-well plate 1 day before the platycodin D treatment. The CellTiter-Glo reagent (equal to the culture volume) was added into each well and incubated at room temperature for 10 min. The bioluminescent signal was determined using GloMax plate Reader (Promega) with CellTiter-Glo assay program. For clonogenic assays, 1,000 cells were seeded onto each well of a 6-well plate and treated with or without 10 mmol/L platycodin D for 5 days. After staining with 1% (w/v) crystal violet, the number of colonies was measured.

### 2.4 Apoptosis assay

Apoptosis assay was performed using the Annexin V–FITC Apoptosis Kit (Biolegend) as described previously. (Wang et al., 2020) The apoptotic cells were measured by a FacsCaibur cell analyzer (BD Biosciences).

### 2.5 Western blot analysis

Western blot analysis was performed as described previously (Wang et al., 2020). The following antibodies were used: Bcl-2 (#4223), Phospho-Bcl-2 (#2875), Bcl-xL (#2764), Bid (#2002), Bax (#5023), Bad (#9239), phospho-JNK (#4668), JNK (#9252), JNK1 (#3708), JNK2 (#9258), Cleaved Caspase-3 (#9664), PARP (#9532), phospho-c-Jun (Ser73) (#3270), phospho-c-Jun (Ser63) (#91952), c-Jun (#9165), PUMA (#4976), GAPDH (SC-47724). The antibodies were used at either 1:1,000 dilution or 1:2000 dilution. The GAPDH antibodies were purchased from Santa Cruz Biotechnology, and the rest of the antibodies were purchased from Cell Signaling Technology. The band intensity of the target protein was quantified using VisionWork LS image acquisition and analysis software (UVP).

### 2.6 Mitochondrial function analyses

The effect of platycodin D on mitochondrial function was analyzed using a Seahorse XFe96 analyzer with FX Cell Mito Stress Test kit according to the manufacturer’s protocol (Seahorse Biosciences). Briefly, 5,000 cells/well were seeded onto each well of a Seahorse 96-well plate and treated with platycodin D (0–15 μmol/L) for 24 h. The OCR (pmol/min/1,000 cells) was measured in real time, with injections of oligomycin (ATP synthase inhibitor), carbonyl cyanide-p-trifluoro-methoxyphenyl-hydrazone (FCCP, mitochondrial uncoupler), and rotenone/antimycin A (respiration inhibitor) to evaluate OCR per 1,000 cells from proton leak, maximal respiration, and nonmitochondrial oxygen consumption, respectively. Mitochondrial SRC = maximal OCR—basal OCR.

### 2.7 Quantitative real-time PCR (qPCR)

The Trizol reagent (Invitrogen) was used to purify the total RNA from the cells. After synthesis of the first-strand cDNA using Superscript First-Strand Kit (Invitrogen), the *PUMA* and *GAPDH* mRNA levels were determined by qPCR using primers purchased from Integrated DNA Technologies. The relative *PUMA* mRNA levels were calculated by the 2−ΔΔCt method with three replicates (Livak and Schmittgen, 2001).

### 2.8 Chromatin immunoprecipitation assay

ChIP assay was performed using ChIP-IT Express kit (Active Motif) as previously described (Wang et al., 2012). The DNA/AP-1 complex was precipitated using c-Jun antibody (#9165, Cell Signaling Technology). The pre-immune rabbit serum was used as the negative control. The immunoprecipitated *PUMA* promoter DNA was detected by PCR using following primers: Forward: 5’-AGA​TTA​CCT​GCA​TCT​CTT​GG-3; Reverse: 5’-CCC​TGC​TCT​GGT​TTG​GTG​AGT-3’.

### 2.9 Transfection and luciferase assay

The *PUMA* promoter (-150 bp to +50 bp relative to transcription start site containing the AP-1 binding site) (Cazanave et al., 2009) was synthesized and cloned into pGL-3 basic vector (Promega). The 4X AP-1 luciferase reporter plasmid was as previously described (Wang et al., 2008). Cells (3 × 10^4^ cells per well in a 24-well plate) were transfected with 0.2 μg of *PUMA* promoter luciferase reporter or 0.2 μg of 4X AP-1 luciferase reporter along with 10 ng of Renilla luciferase reporter (pRL-SV40) using PolyJet transfection reagent (SignaGen Laboratories). Cells were lysed in 1 × passive buffer (Promega) and the bioluminescent signal was determined with 20/20 luminometer using Dual-Luciferase Reporter Assay kit (Promega).

### 2.10 Knockdown of PUMA and JNK

Cells (2 × 10^5^ cells on a 60-mm plate) were transfected with 110 pmol of scrambled, *JNK1*, *JNK2*, or *PUMA* siRNA using INTERFERin *in vitro* siRNA transfection reagent (Polyplus) according to the manufacturer’s protocol. The *JNK1* (sc-29380), *JNK2* (sc-39101), and *PUMA* (sc-37153) siRNAs were purchased from Santa Cruz Biotechnologies. Forty-8 hours post-transfection, cells were harvested for downstream applications or Western blot analyses.

### 2.11 *In vivo* xenograft study

The animal protocol of the present study was approved by the Ethical Committee of the Guang’anmen Hospital, China Academy of Chinese Medical Sciences. NOD/scid nude mice (4- to 5-week old) were purchased from Charles River and housed under pathogen-free conditions with a commercial diet, water *ad libitum*, and 12 h light/12 h dark cycle. H1299 cells (1 × 10^7^ cells) were injected s.c. into the flank of each mouse and the tumor size was determined as previously described (Zhang et al., 2015). Mice were randomly divided into two groups (6 mice per group) when tumors reached 50–100 mm^3^. One group of mice was treated with vehicle [i.p. injection of 10% (v/v) DMSO in corn oil] and the other group of mice was i.p. injected with platycodin D (8 mg/kg) every day for 14 days. All mice under these treatments showed no sign of toxicity, i.e., bodyweight loss of more than 15%, diarrhea, or decreased food intake.

### 2.12 Immunohistochemical analyses

The tissue samples were fixed with 4% neutral buffered paraformaldehyde, embedded in paraffin, and sectioned into 4-μm slices. Tissue sections were de-waxed by heating at 60°C, washed in xylene, and re-hydrated through a graded series of ethanol and water. After antigen retrieval, inactivation of endogenous peroxidase with 3% H_2_O_2_, and block of tissues with normal goat serum, the tissue sections were incubated with antibodies against Ki-67 (ab92742, 1:500 dilution, Abcam), Cleaved Caspase-3 (ab 2,302, 1:500 dilution, Abcam), PUMA (#4976, 1:100 dilution, Cell Signaling), phospho-JNK (#4668, 1:100 dilution, Cell Signaling), JNK (#9252, 1:100 dilution, Cell Signaling), and phospho-c-Jun (Ser63) (#91952, 1:100 dilution, Cell Signaling), followed by biotin-linked secondary antibody using Dako REAL EnVision Detection System (K5007, DAKO). Three random areas of the tumor slide were quantified using Image-pro plus 6.0 software (Media Cybernetics, Inc.).

### 2.13 Statistical analysis

Differences in the band intensity of the Western blot were analyzed using the one-sample t-test. The differences control group and the experimental group were analyzed through the two-sample t-test. Data are shown as the mean ± standard deviation. The difference is considered statistically significant at the level of *p* < 0.05.

## 3 Results

### 3.1 Platycodin D inhibits cell viability and proliferation in lung cancer cells

**FIGURE 1:**
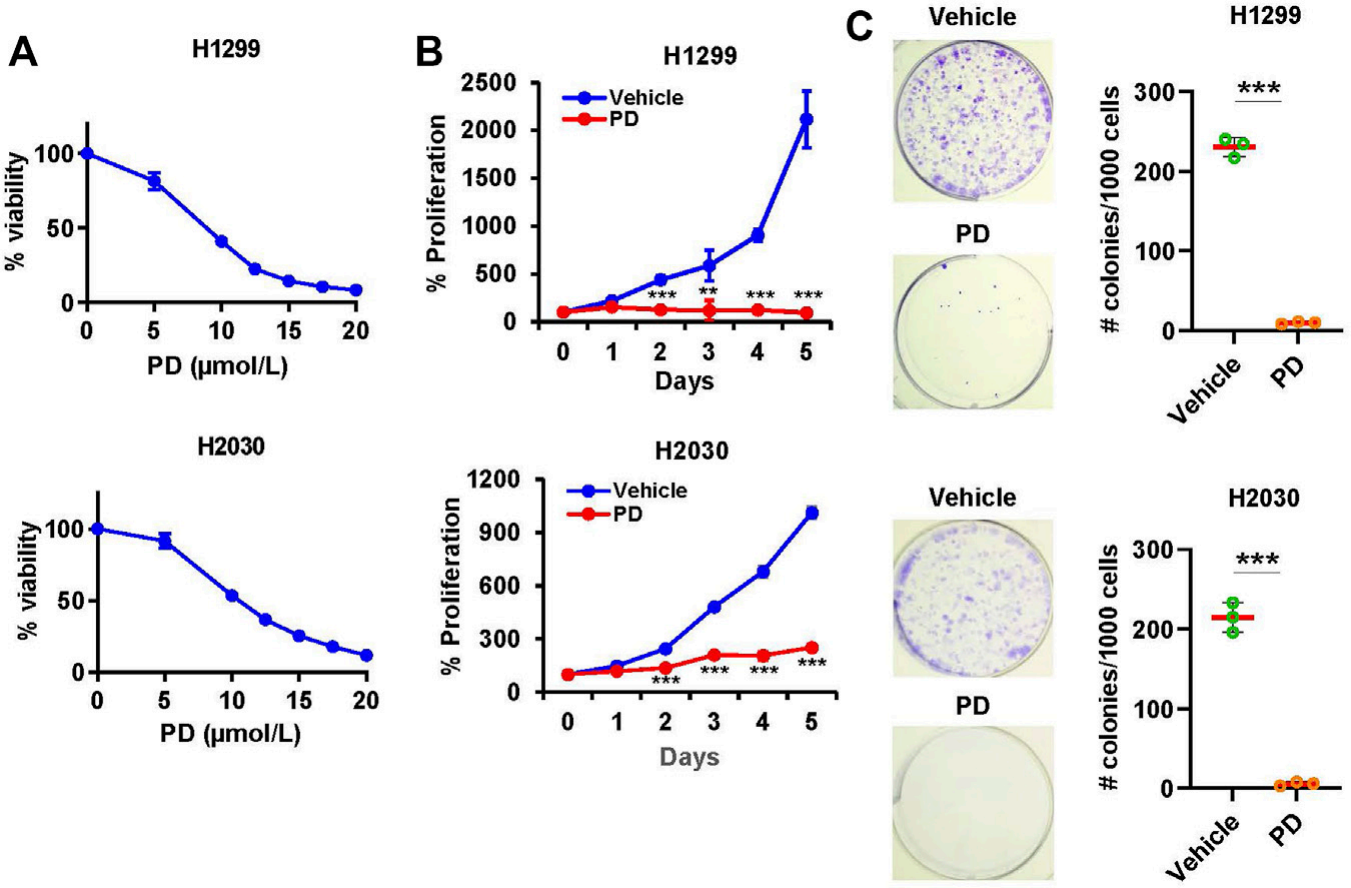
Platycodin D inhibits cell viability and colony formation in lung cancer cells. **(A)** Cells were treated with platycodin D (0–20 μmol/L) for 48 h and determined by CellTiter-Glo® Luminescent Cell Viability Assay kit. The bioluminescent signal at 0 μmol/L is designated as 100%. Results are expressed as mean ± SD (*n* = 4). **(B)** Cells were treated with vehicle or platycodin D (10 μmol/L) for 5 days and assessed using CellTiter-Glo® Luminescent Cell Viability Assay kit. The bioluminescent signal at day 0 is designated as 100%. Data were analyzed using two-sample *t*-test (mean ± SD, *n* = 4, **p < 0.01; ***p < 0.001). **(C)** Cells were treated with vehicle or platycodin D (10 μmol/L) for 5 days. Data were analyzed by a two-sample *t*-test (mean ± SD, *n* = 3, ***p < 0.001). PD: platycodin D.

To examine the effect of Platycodin D on cell viability in NSCLC, H1299, H2030, and A549 cells were treated with Platycodin D from 0 to 20 μmol/L for 48 h. We found that Platycodin D inhibited the viability of lung cancer cells in a dgose-dependent manner (Figure 1A and Supplementary Figure S1). The IC_50_ value of Platycodin D for H1299, H2030, and A549 cells was 7.8 μmol/L, 9.6 μmol/L, and 10.3 μmol/L, respectively (Supplementary Figure S2). To examine whether Platycodin D inhibits proliferation, H1299 and H2030 cells were treated with Platycodin D at 10 μmol/L for 5 days. We found that Platycodin D significantly inhibited the viability of H1299 and H2030 cells, approximately 95% and 75% at day 5, respectively, compared to vehicle-treated cells (Figure 1B). Results of clonogenic survival assays also indicated that cells treated with 10 μmol/L of Platycodin D significantly reduced the colony number (Figure 1C). The colony number of H1299 and H2030 treated with 10 μmol/L of Platycodin D for 5 days was about 5% and 2%, respectively, comparing with that of vehicle-treated cells. Taken together, these results demonstrated that Platycodin D exhibits anti-proliferative activity in NSCLC.

### 3.2 Platycodin D induces apoptosis in lung cancer cells

**FIGURE 2:**
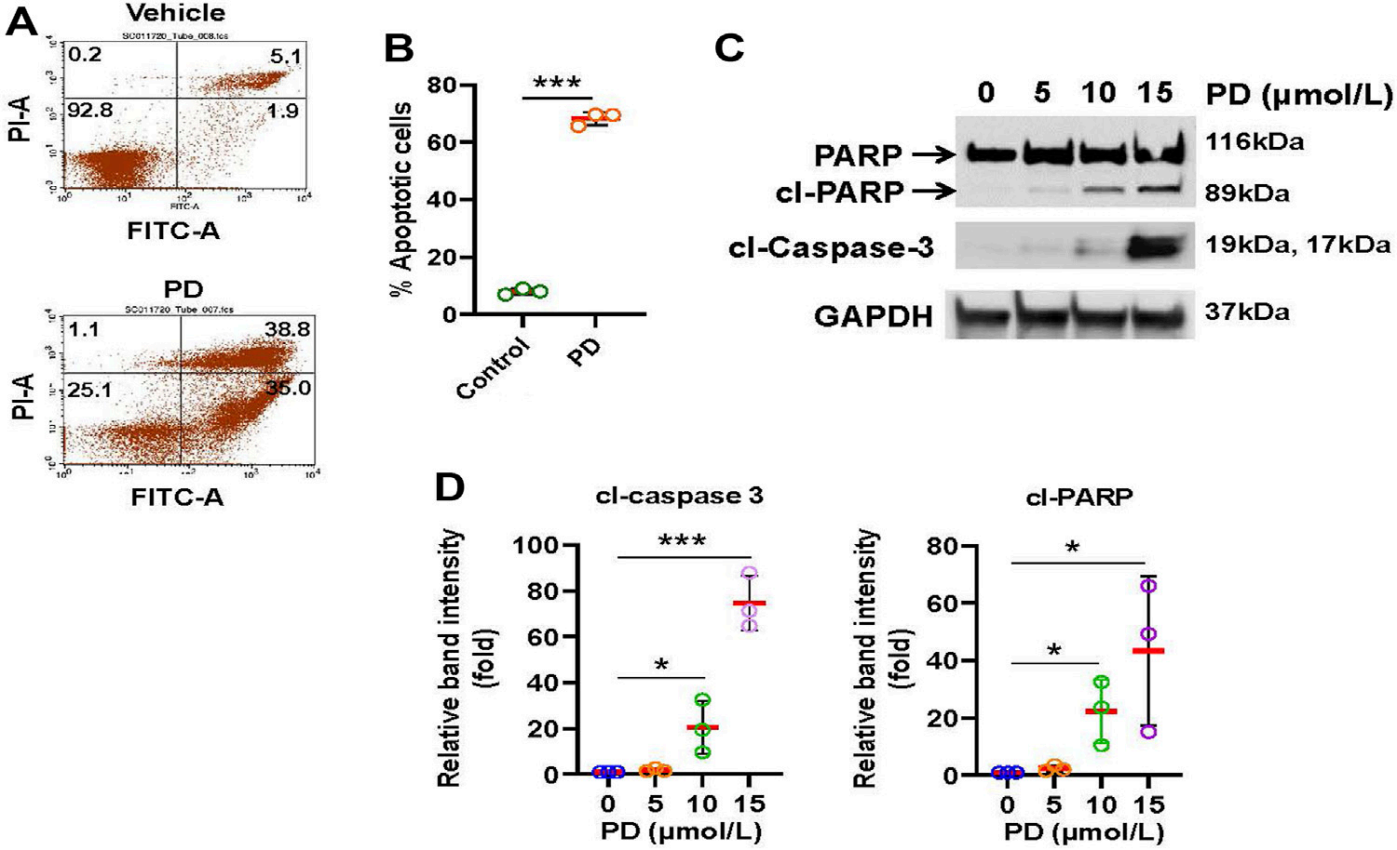
Platycodin D induces apoptosis in lung cancer cells. **(A)** H1299 cells were treated with platycodin D (15 μmol/L) for 48 h and the apoptotic cells were determined by Annexin V-FITC/PI flow cytometry. The represented histograms are shown. The number in each quadrant represents the percentage of total cells. **(B)** Quantification of the apoptotic [early (Annexin V+/PI-) and late (Annexin V+/PI+)] cells in **(A)**. Data were analyzed using two-sample t-test (mean ± SD, *n* = 3, ***p < 0.001). **(C)** H1299 cells were treated with 0–15 μmol/L of Platycodin D for 48 h and subsequently used for Western blot analyses. **(D)** Densitometric quantification of cleaved PARP and cleaved caspase 3 (19 kDa + 17 kDa) levels in **(C)**. The ratio of cleaved PARP/GAPDH or cleaved caspase 3/GAPDH in vehicle-treated (0 μmol/L) cells is designated as 1. Data were analyzed using one-sample *t*-test (mean ± SD, *n* = 3, *p < 0.05; ***p < 0.001). PD: platycodin D; cl-PARP: cleaved PARP; cl-caspase 3: cleaved caspase.

Next, we explored whether Platycodin D inhibits cell viability through the induction of apoptosis. H1299 cells were treated with Platycodin D and subsequently stained with Annexin V-FITC and Propidium Iodide. The apoptotic cells were then determined by flow cytometry. As shown in Figures 2A,B, apoptotic cells increased approximately 8-fold in Platycodin D-treated cells than those in vehicle-treated cells. In addition, the protein levels of apoptosis markers, cleaved poly(ADP-ribose) polymerase (PARP) and cleaved caspase-3, increased remarkably when cells treated with 10 and 15 μmol/L of Platycodin D for 48 h (Figures 2C,D). Similar results were also observed when H2030 cells were treated with Platycodin D (Supplementary Figure S3). These data indicated that Platycodin D induces apoptosis in lung cancer cells.

### 3.3 Platycodin D impairs mitochondrial function

**FIGURE 3:**
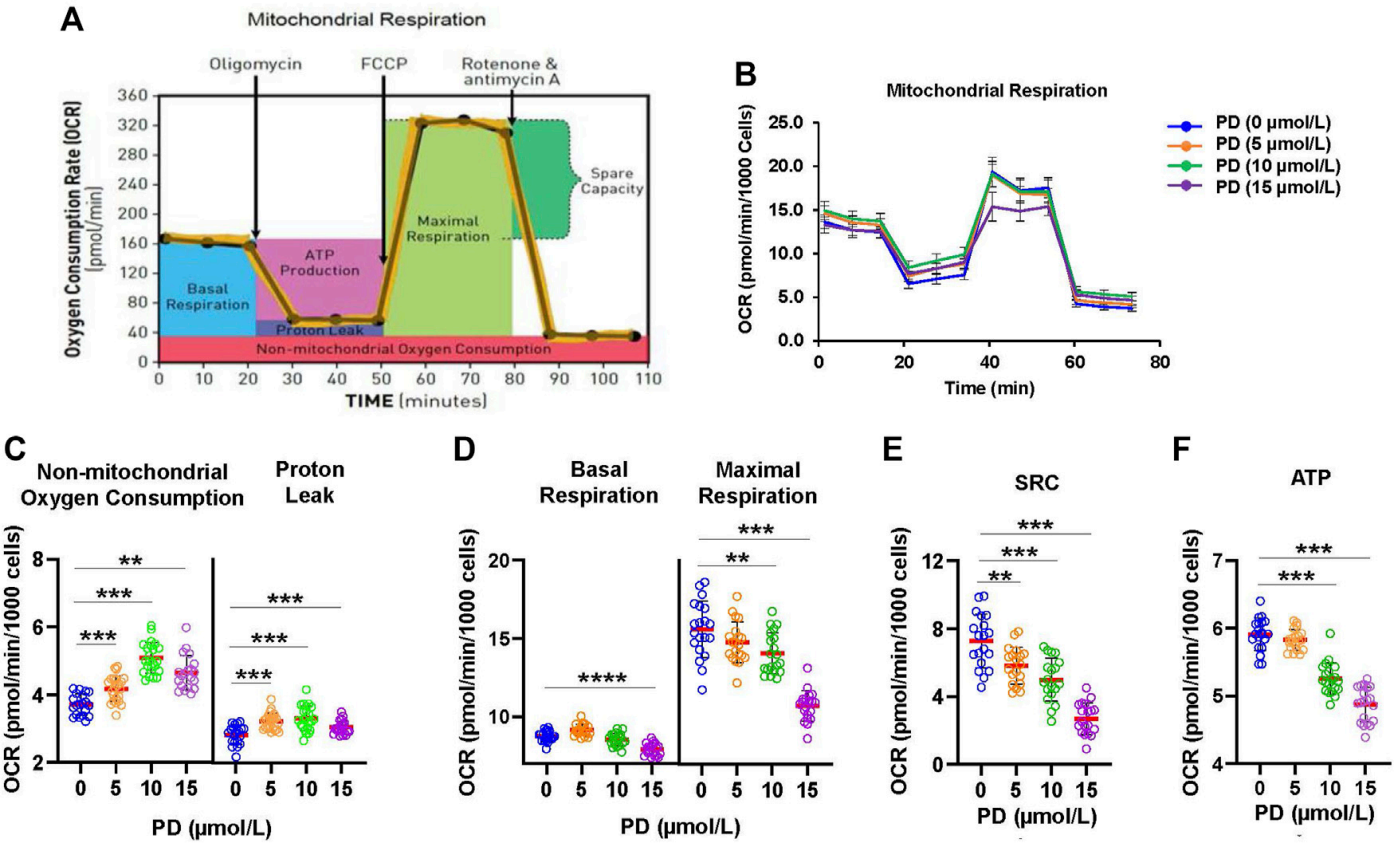
Platycodin D promotes mitochondrial dysfunction. **(A)** The schematic diagram for mitochondrial function analysis using a Seahorse XFe96 analyzer. After determining basal OCR, oligomycin, FCCP, and rotenone/antimycin A were sequentially injected to measure OCR from proton leak, maximal respiration, and nonmitochondrial oxygen consumption, respectively. OCR of nonmitochondrial respiration was subtracted from OCR at each stage to calculate the net OCR for basal respiration, proton leak, and maximal respiration values. **(B)** H1299 cells were treated with 0–15 μmol/L of platycodin D for 24 h and subjected to mitochondrial function analysis using a Seahorse XFe96 analyzer. **(C)** The OCR values of proton leak and non-mitochondrial oxygen consumption in **(B)**. **(D)** The OCR values of basal and maximal respiration in **(B)**. **(E)** The mitochondrial SRC was calculated from **(B)** using the formula: maximal respiration OCR—basal respiration OCR. **(F)** The ATP production was calculated from **(B)** using the formula: basal respiration OCR—proton leak OCR. Data were analyzed using two-sample *t*-test (mean ± SD, *n* = 20, **p < 0.01; ***p < 0.001). PD: platycodin D.

Release of cytochrome c from the mitochondria to cytosol is a devoting step for apoptosis. Cytochrome c is the electron carrier in the mitochondrial electron transport chain. Thus, disruption of the electron transport chain has been recognized to be an early event for apoptosis (Green and Reed, 1998). To evaluate the effects of platycodin D treatment on mitochondrial function, we analyzed the oxygen consumption rate (OCR) in H1299 cells with platycodin D treatment (0–15 μmol/L) using the Seahorse analyzer (Figure 3B). Non-mitochondrial respiration and proton leak were slightly increased by platycodin D treatment (Figure 3C). However, the basal and maximal respiration decreased when cells were treated with 10 and 15 μmol/L platycodin D (Figure 3D). The spare respiratory capacity (SRC) value is defined as the difference between basal OCR production and its maximal OCR, which is a parameter for the function of mitochondria (Hill et al., 2012). The reduction of SRC value suggests the onset of apoptosis of cells (Marchetti et al., 2020). In agreement with platycodin D-induced apoptosis, platycodin D treatment decreased SRC and ATP production in a dose-dependent manner (Figures 3E,F). Cells treated with 15 μmol/L of platycodin D for 24 h significantly reduced the values of SRC and ATP production from approximately 7.3 and 5.9 pmol/min/1,000 cells to 2.7 and 4.9 pmol/min/1,000 cells, respectively. Our results indicated that cells treated with platycodin D impaired the electron transport chain and reduced ATP production.

### 3.4 Platycodin D stimulates PUMA expression

**FIGURE 4:**
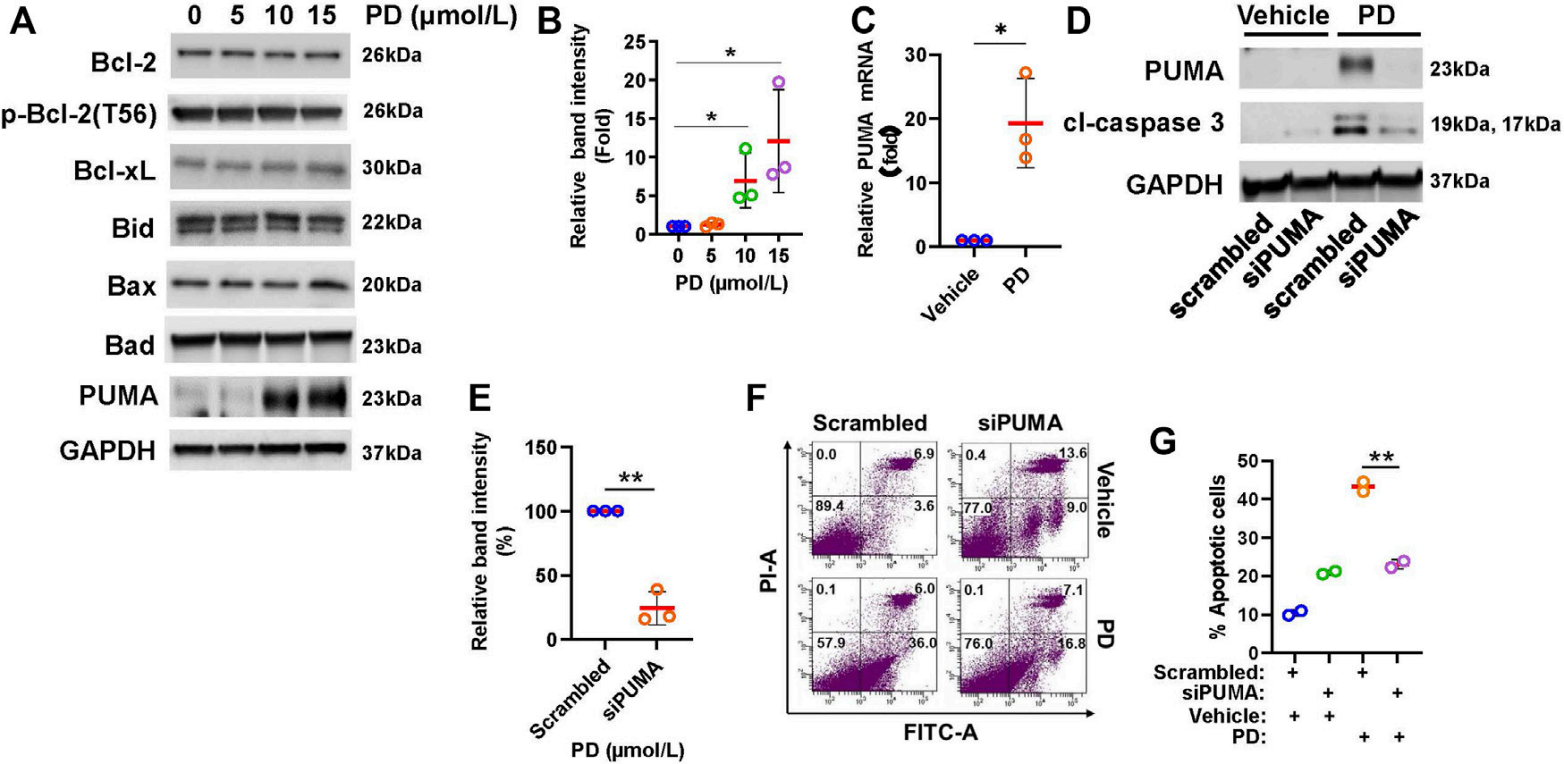
Platycodin D up-regulates PUMA expression. **(A)** H1299 cells were treated with 0–15 μmol/L of platycodin D for 48 h and subjected to Western blot analyses of Bcl-2-related proteins. **(B)** Densitometric quantification of PUMA levels in **(A)**. The ratio of PUMA/GAPDH in vehicle-treated (0 μmol/L) cells is designated as 1. Data were analyzed using one-sample *t*-test (mean ± SD, *n* = 3, *p < 0.05). **(C)** Platycodin D up-regulated *PUMA* mRNA. H1299 cells were treated with platycodin D (15 μmol/L) for 24 h and *PUMA* mRNA was quantified with RT-qPCR. The value of the cells treated with the vehicle is designated as 1. Data were analyzed using one-sample *t*-test (mean ± SD, *n* = 3, *p < 0.05). **(D)** Knockdown of PUMA suppressed platycodin D-induced cleaved caspase 3. H1299 cells were transfected with scrambled siRNA or *PUMA* siRNA. Four-8 hours post-transfection, cells were treated with 15 μmol/L of platycodin D for an additional 24 h. The cells were then used for Western blot analyses. **(E)** Densitometric quantification of cleaved caspase 3 (19 kDa + 17 kDa) levels in **(D)**. The ratio of cleaved caspase 3/GAPDH in scrambled and platycodin D-treated cells is designated as 100%. Data were analyzed using one-sample *t*-test (mean ± SD, *n* = 3, *p < 0.05). **(F)** Knockdown of PUMA attenuates platycodin D-induced apoptosis. H1299 cells were transfected with scrambled siRNA or *PUMA* siRNA. Four-8 hours post-transfection, the cells were treated with vehicle or platycodin D (15 μmol/L) for an additional 24 h. The apoptotic cells were analyzed using Annexin V-FITC/PI flow cytometry. The represented histograms are shown. The number in each quadrant represents the percentage of total cells. **(G)** Quantification of the apoptotic [early (Annexin V+/PI-) and late (Annexin V+/PI+)] cells in **(E)**. Data were analyzed using two-sample t-test (mean ± SD, *n* = 2, **p < 0.01). PD: platycodin D.

To understand how Platycodin D induces mitochondrial dysfunction and apoptosis in lung cancer cells, we examined the expression level of anti-apoptotic and pro-apoptotic proteins of the Bcl-2-related proteins using Western blotting analysis. As shown in Figure 4A, we found that H1299 cells treated with up to 15 μmol/L of Platycodin D did not alter the level of anti-apoptotic proteins, Bcl-2, phosphorylated Bcl-2 [Bcl-2 phosphorylation may enhance apoptosis (De Chiara et al., 2006)], and Bcl-xl. Platycodin D also did not change the expression of pro-apoptotic proteins, Bax, Bid, and Bak. In contrast, Platycodin D induced the PUMA protein expression in a dose-dependent manner (Figures 4A,B). The PUMA protein level in the 15 μmol/L of Platycodin D-treated H1299 cells was approximately 12-fold compared to that in vehicle-treated cells. Next, we examined whether Platycodin D alters *PUMA* mRNA levels. H1299 cells were treated with 15 μmol/L of Platycodin D for 24 h and the *PUMA* mRNA level was determined using RT-qPCR. The level of *PUMA* mRNA was approximately 19-fold higher in platycodin D-treated cells than that in vehicle-treated cells (Figure 4C), indicating that Platycodin D up-regulated PUMA at the transcriptional level.

To test whether induction of PUMA expression is a key event for platycodin D-induced apoptosis, cells were knocked down PUMA and subsequently treated with platycodin D at 15 μmol/L for 24 h. As shown in Figures 4D,E, platycodin D treatment dramatically increased the level of the apoptosis marker, cleaved caspase 3, in cells transfected with scrambled siRNA. However, the *PUMA* knockdown attenuated the increase of platycodin D-induced cleaved caspase 3. Knockdown of PUMA in H2030 cells also repressed platycodin D-induced cleaved caspase 3 (Supplementary Figure S4). In agreement with reducing cleaved caspase 3 by PUMA knockdown, knockdown of PUMA significantly attenuated platycodin D-induced apoptosis in H1299 cells (Figures 4F,G). These findings indicated that up-regulation of PUMA contributed to platycodin D-induced apoptosis in NSCLC cells.

### 3.5 Platycodin D up-regulates PUMA through JNK1/AP-1 pathway

**FIGURE 5:**
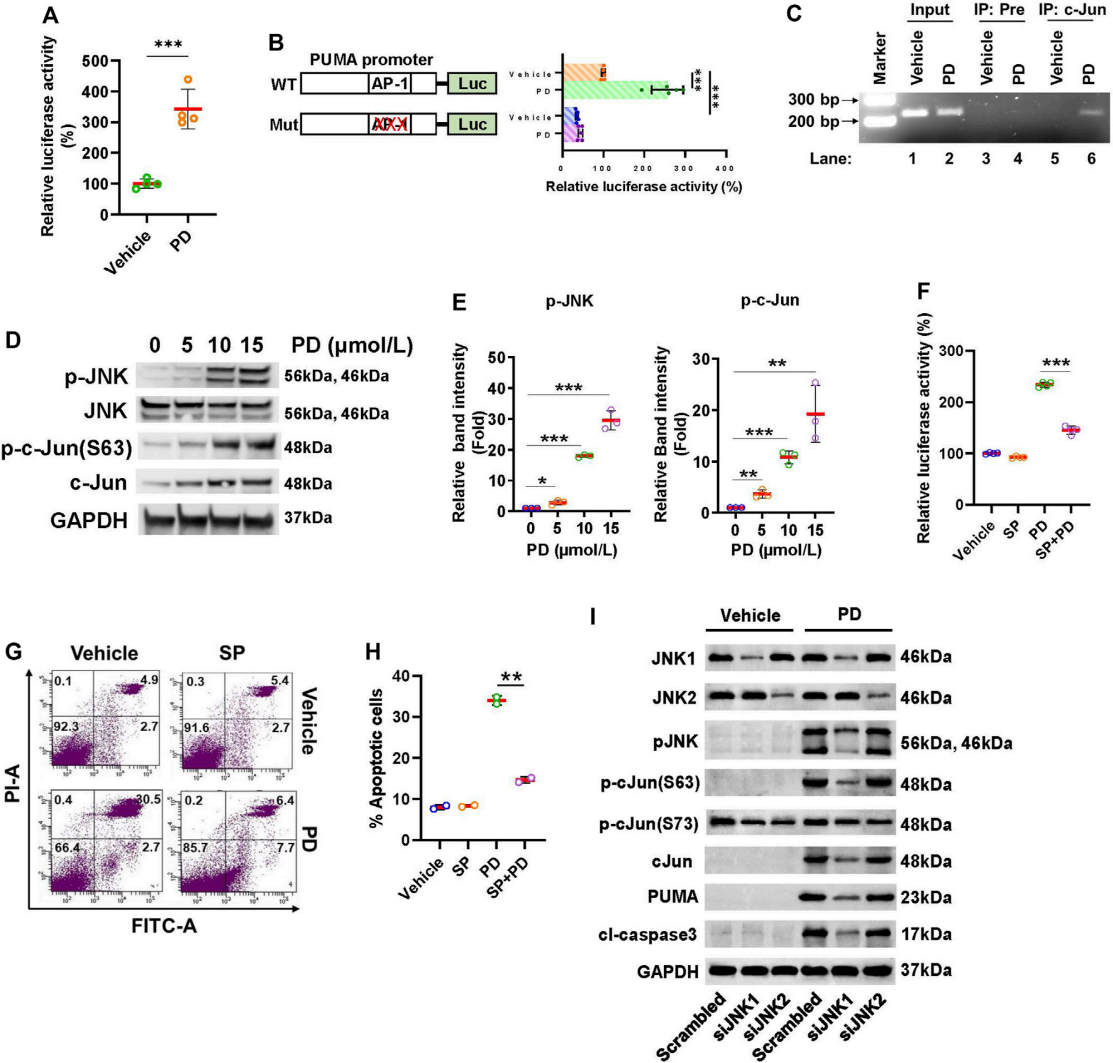
Platycodin D activates the JNK1/AP-1 axis to up-regulate PUMA. **(A)** Platycodin D activates AP-1 dependent transcription. Cells were transfected with 4 × AP-1 luciferase reporter and subjected to platycodin D (15 μmol/L) treatment. The average luciferase activity with vehicle-treated cells is designated as 100%. Data were analyzed by two-sample *t*-test (mean ± SD, *n* = 4, ***p < 0.001). **(B)** Platycodin D stimulates PUMA promoter activity. The H1299 cells were transfected with PUMA-luciferase plasmid (WT) or AP-1 binding site mutated PUMA-luciferase plasmid (Mut) and subjected to platycodin D (15 μmol/L) treatment. The average luciferase activity in cells transfected with WT without platycodin D treatment is designated as 100%. Data were analyzed using two-sample *t*-test (mean ± SD, *n* = 5, ***p < 0.001). **(C)** Platycodin D stimulates AP-1 binding to the PUMA promoter. Cells were treated with vehicle or 15 μmol/L of platycodin D for 24 h and were subsequently used for ChIP assays using preimmune IgG or c-Jun antibody. The DNAs from cell lysate (input) and ChIP enriched were amplified by PCR using primers as indicated in the Materials and Methods. The representative PCR products were resolved onto 3% agarose gels. **(D)** Platycodin D increases the phosphorylation of JNK and c-Jun. Cells were treated with 0–15 μmol/L of platycodin D for 48 h. Western blot was performed using the antibodies as indicated. **(E)** Densitometric quantification of phospho-JNK and phospho-c-Jun(Ser63) levels in **(D)**. The ratio of phospho-JNK/GAPDH or phospho-c-Jun(Ser63)/GAPDH in vehicle-treated (0 μmol/L) cells is designated as 1. Data were analyzed by one-sample *t*-test (mean ± SD, *n* = 3, *p < 0.05; **p < 0.01; ***p < 0.001). **(F)** Inhibition of JNK attenuates platycodin D-induced AP-1 activation. The wild-type PUMA-luciferase plasmid was transfected into H1299 cells. Cells were treated with vehicle (DMSO) or SP600125 (20 µM) for 1 h and subsequently treated with vehicle or platycodin D (15 μmol/L) for an additional 6 h. The average luciferase activity with vehicle-treated cells is designated as 100%. Data were analyzed by two-sample *t*-test (mean ± SD, *n* = 4, ***p < 0.001). **(G)** Inhibition of JNK attenuates platycodin D-induced apoptosis. H1299 cells were treated with vehicle (DMSO) or SP600125 (20 µM) for 1 h and then treated with vehicle or platycodin D (15 μmol/L) for an additional 24 h. The apoptotic cells were analyzed using Annexin V-FITC/PI flow cytometry. The represented histograms are shown. The number in each quadrant represents the percentage of total cells. **(H)** Quantification of the apoptotic [early (Annexin V+/PI-) and late (Annexin V+/PI+)] cells in **(G)**. Data were analyzed by two-sample *t*-test (mean ± SD, n = 2, **p < 0.01). **(I)** Knockdown of JNK1 but not JNK2 reduces PUMA and cleaved caspase 3 levels. Cells were transfected with scrambled siRNA, *JNK1* siRNA, or *JNK2* siRNA and subsequently treated with platycodin D (15 μmol/L) for 24 h. Western blot was performed using antibodies as indicated. PD: platycodin D; SP: SP600125; Pre: pre-immune IgG; cl-caspase 3: cleaved caspase 3.

It has been reported that the AP-1 transcription factor binds to the *PUMA* promoter and stimulates PUMA expression (Cazanave et al., 2009). To test whether Platycodin D activates AP-1 dependent transcription in lung cancer cells, H1299 cells were transfected with 4× AP-1 reporter followed by platycodin D (15 μmol/L) treatment for 24 h. As shown in Figure 5A, the AP-1 dependent transcription was significantly stimulated by platycodin D treatment. To investigate whether AP-1 mediates platycodin D-induced PUMA expression, the PUMA promoter (-150 bp to +50 bp relative to the transcription start site containing an AP-1 binding site located from -14 bp to -8 bp, Supplementary Figure S5) (Cazanave et al., 2009) was cloned and fused with a luciferase reporter. The PUMA-luciferase plasmid was then transfected into H1299 cells and subsequently treated with vehicle or platycodin D (15 μmol/L) for 24 h. The luciferase activity in platycodin D-treated cells was approximately 2.5-fold of that seen in vehicle-treated cells (Figure 5B). However, the basal and platycodin D-induced *PUMA* promoter activity was dramatically reduced when the AP-1 binding site was mutated (Figure 5B). To further confirm that AP-1 regulates PUMA transcription, chromatin immunoprecipitation (CHIP) assays were performed to verify the binding of AP-1 to the *PUMA* promoter. The H1299 cells were treated with platycodin D (15 μmol/L) for 24 h and the DNA/AP-1 complex was precipitated with a c-Jun antibody. The *PUMA* promoter was then detected from the precipitated DNAs by PCR using *PUMA* primers, which amplified the AP-1 binding site at -14 to -8 bp from the transcription start site (Cazanave et al., 2009). As shown in Figure 5C, the input chromatin and pre-immune serum were used as the positive and negative control, respectively (lanes 1–4). The PCR product (229 bp) was observed using c-Jun antibody precipitated chromatin from lysates of platycodin D-treated cells (lane 6). In contrast, this PCR product was not detected in the cell lysates of vehicle-treated cells (lane 5). Taken together, these findings indicated that platycodin D activates AP-1 and promotes PUMA transcription.

Next, we examine whether platycodin D activates JNK since JNK is the key kinase to activate c-Jun, the major component of AP-1. H1299 cells were treated with 0–15 μmol/L of platycodin D and the levels of total and phosphorylated JNK were assessed. Platycodin D treatment increased the levels of phosphorylated JNK but not total JNK in a dose-dependent manner (Figures 5D,E). In agreement with the activation of AP-1 by platycodin D, the levels of both total and phosphorylated c-Jun at Ser63 were increased by platycodin D treatment. The increase in the total c-Jun protein might be due to c-Jun positive autoregulation (Angel et al., 1988). To further define the role of the JNK/AP-1 axis in the regulation of PUMA expression, the PUMA-luciferase reporter was transfected into H1299 cells and subsequently treated with the JNK inhibitor, SP600125 (20 µM) for 1 h in prior to platycodin D (15 μmol/L) treatment (Liu et al., 2020). As shown in Figure 5F, the luciferase activity in platycodin D-treated cells was increased compared to that in vehicle-treated cells, while this induction was significantly inhibited by SP600125 treatment, suggesting that inhibition of JNK suppressed platycodin D-induced PUMA expression. Inhibition of JNK activity by SP600125 also showed to reduce the platycodin D-induced apoptosis (Figures 5G,H). Taken together, our findings suggested that platycodin D induces apoptosis through the JNK/AP-1 axis.

JNK contains three isoforms, i.e., JNK1, JNK2, and JNK3. The JNK1 and JNK2 are ubiquitously expressed in the tissues throughout the body, but the JNK3 is only expressed in the brain, heart, and testes (Davis, 2000). To find out which JNK isoform is involved in platycodin D-induced c-Jun activation and PUMA expression, we knocked down the JNK1 and JNK2 using *JNK1* and *JNK*2 siRNA, respectively, and subsequently treated with or without platycodin D (15 μmol/L) for 24 h. As shown in Figure 5F and Supplementary Figure S6, the *JNK1* and *JNK2* siRNA successfully knocked down JNK1 and JNK2, respectively, in both platycodin D-treated and vehicle-treated cells. JNK1 knockdown decreased approximately 90% of phospho-JNK and phospho-c-Jun (Ser63) in the platycodin D-treated cells. By contrast, knockdown of JNK2 showed a slight decrease in JNK phosphorylation and no effect on c-Jun phosphorylation at Ser63. In addition, knockdown of JNK1 but not JNK2 significantly decreased approximately 85% and 90% of platycodin D-induced PUMA and cleaved caspase 3 expression, respectively, suggesting that platycodin D-regulated PUMA expression through JNK1. Interestingly, cells treated with platycodin D did not alter the phosphorylation of c-Jun at Ser73 (vehicle-treated scrambled cells vs. platycodin D-treated scrambled cells) and knockdown of JNK1 and JNK2 showed a similar level of reduction in phospho-c-Jun at Ser 73, suggesting that phosphorylation of c-Jun at Ser73 does not mediate the platycodin D-induced PUMA expression.

### 3.6 Platycodin D suppresses tumor growth in mice

**FIGURE 6:**
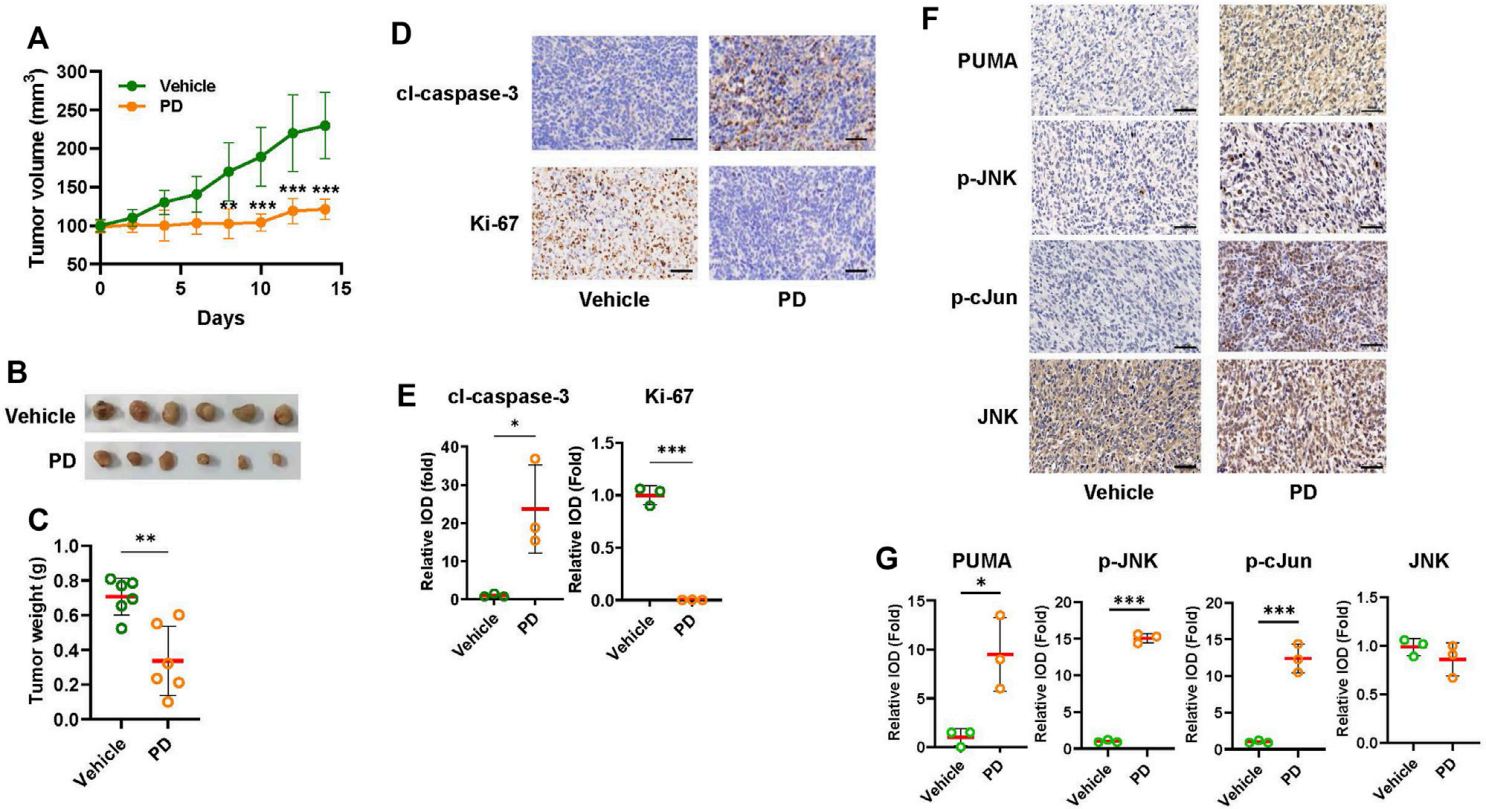
Platycodin D significantly suppresses tumor growth in the H1299 lung xenograft model. NOD nude mice (six mice per group) were treated with vehicle [10% (v/v) DMSO in corn oil] or 8 mg/kg of Platycodin D for 14 days. **(A)** Platycodin D treatment decreased the tumor volume. The tumor volume was measured every other day with calipers. The tumor volume with vehicle-treated tumors at day 0 is designated as 100%. Data were analyzed using two-sample *t*-test (mean ± SD, *n* = 6, **p < 0.01, ***p < 0.001). **(B)** Image of tumors after treatment with the vehicle or platycodin D for 14 days. **(C)** Platycodin D treatment lowers the tumor weight. Data were analyzed by two-sample *t*-test (mean ± SD, *n* = 6, **p < 0.01). **(D)** Platycodin D treatment increased apoptosis in tumors. The expressions of cleaved caspase 3 (apoptosis) or Ki-67 (proliferation) in tumors were examined by IHC staining. The representative images are shown. Bar: 50 μm. **(E)** Quantifications of the cleaved caspase 3 and Ki-67 staining. The average integrated optical density (IOD) in the tumors from vehicle-treated mice is designated as 100%. Data were analyzed by two-sample *t*-test (mean ± SD, *n* = 3, *p < 0.05; ***p < 0.001). **(F)** Platycodin D treatment increased PUMA expression in tumors. The expressions of PUMA, JNK, phospho-JNK, or phospho-c-Jun at Ser 67 in tumors were examined by IHC staining. Bar: 50 μm. **(G)** Quantifications of the PUMA, JNK, phospho-JNK, and phospho-c-Jun(Ser63) staining. The average IOD in tumors from vehicle-treated mice is designated as 100%. Data were analyzed by two-sample *t*-test (mean ± SD, *n* = 3, *p < 0.05; ***p < 0.001). PD: platycodin D; cl-caspase 3: cleaved caspase 3.

To validate the results of the cultured cell study, we tested the efficacy of platycodin D on suppressing tumor growth in H1299 cells-derived tumor xenograft. The tumor growth in mice treated with platycodin D (8 mg/kg) was slower than those treated with vehicle (Figure 6A). The average tumor volume in the platycodin D-treated mice was approximately 50% of that in the vehicle-treated mice (121 mm^3^ vs. 230 mm^3^, *p* < 0.001) at the end of 14-day treatment. In agreement with the results of tumor volume, the average tumor weight in the platycodin D-treated mice was approximately 50% of that in the vehicle-treated mice (Figures 6B,C). To demonstrate that platycodin D stimulates apoptosis *in vivo*, the tumor tissue sections were stained with antibodies against Ki-67 and cleaved caspase-3 to evaluate the proliferation and apoptosis, respectively (Figure 6D). The tumor tissues from platycodin D-treated mice showed a significant increase in cleaved caspase 3 staining than those from vehicle-treated mice (Figure 6E). Conversely, the Ki-67 positive-staining cells in the tumors from platycodin D-treated mice was dramatically reduced compared to those from vehicle-treated mice (Figure 6E). To confirm the *in vitro* observation that platycodin D induces apoptosis through up-regulation of PUMA, the tumor tissues were further immunohistochemical stained with antibodies against PUMA, JNK, phospho-JNK, and phospho-c-Jun(Ser63). As shown in Figures 6F,G, the tumor tissues from platycodin D-treated mice showed a significant increase in staining of PUMA, phospho-JNK, and phospho-c-Jun(Ser63) compared to those from vehicle-treated mice, but the total JNK staining in platycodin D-treated tumors and vehicle-treated tumors is similar. We also noted that the JNK was mainly located in the cytoplasm in the tumors from vehicle-treated mice while it was mainly located in the nucleus in the tumors from platycodin D-treated mice, indicating that JNK is activated by platycodin D since JNK activation leads to JNK nuclear translocation (Mizukami et al., 1997). These findings indicated that platycodin D up-regulates PUMA expression and activates JNK and AP-1 *in vivo*.

## 4 Discussion

**FIGURE 7:**
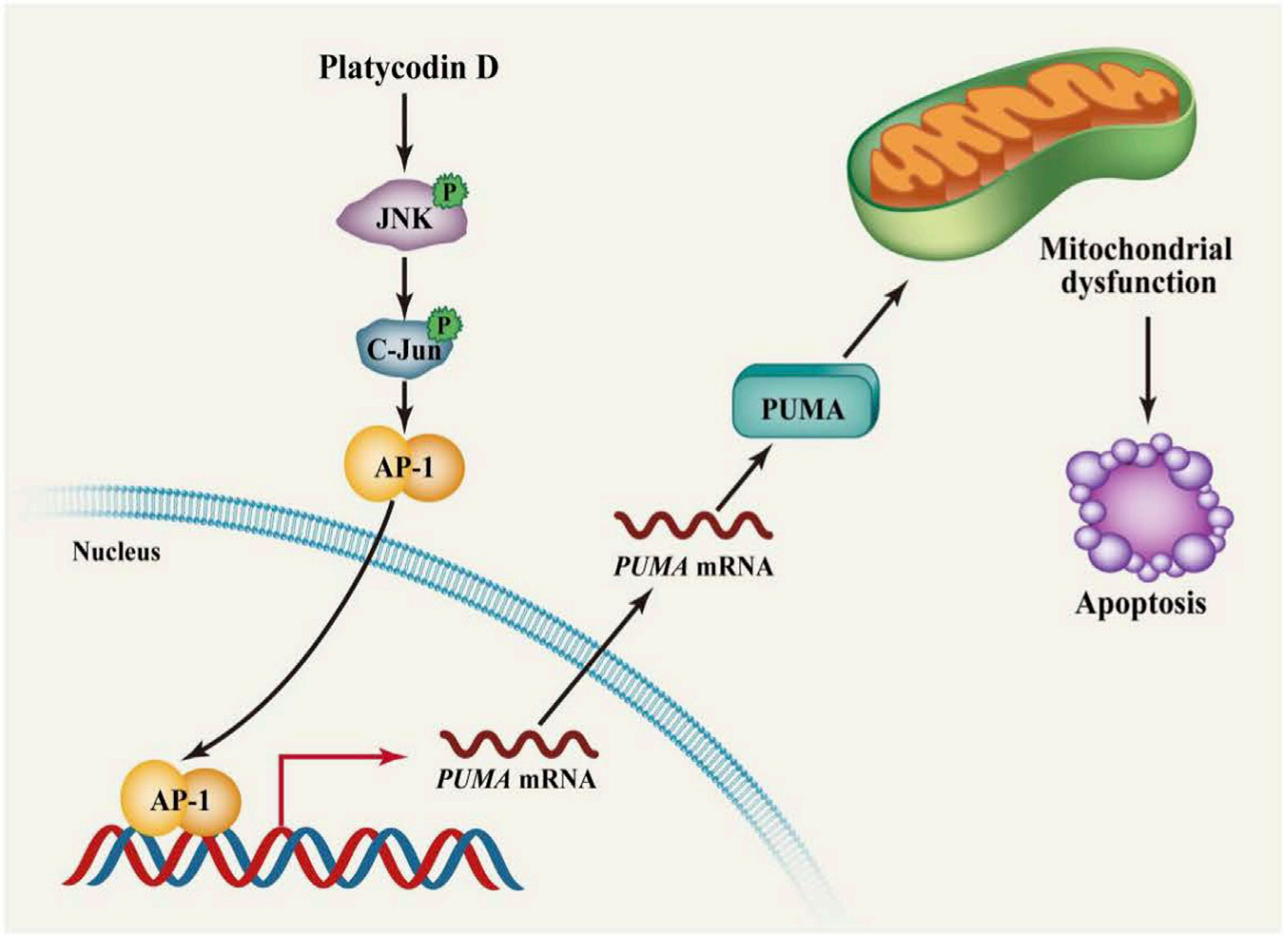
Schematic diagram showing that platycodin D induces PUMA expression *via* the JNK/AP-1 pathway to promote apoptosis. Platycodin D activates JNK1 leading to the translocation of JNK1 into the nucleus. The nuclear JNK1 phosphorylates c-Jun at Ser 63 resulting in the activation of AP-1 transcription factor. The AP-1 transcription factor then binds to the *PUMA* promoter to stimulate PUMA transcription. Up-regulation of PUMA leads to impairment of mitochondrial function, eventually inducing apoptosis.

Although it has been reported that platycodin D treatment induces apoptosis in cells, the role of pro-apoptotic PUMA in platycodin D-induced apoptosis has not been reported. In this study, we discover that up-regulation of PUMA by platycodin D is the critical event for platycodin D-induced apoptosis. Our data showed that platycodin D stimulated the expression of PUMA and cleaved-caspase 3, while PUMA knockdown decreased the platycodin D-induced cleaved-caspase 3 level and apoptosis (Figure 4). Cells treated with platycodin D resulted in activation of JNK1, which further activated AP-1 transcription factor and led to AP-1 binding to PUMA promoter to stimulate its transcription (Figure 5). These molecular events were further confirmed by immunohistochemical staining of the tumors from platycodin D-treated mice (Figure 6). Thus, our findings for the first time to provide mechanistic insight into how platycodin D up-regulates PUMA to induce apoptosis *via* JNK1/AP-1 pathway (Figure 7).

Platycodin D appears to induce apoptosis and/or growth arrest in various cancer cells (Khan et al., 2016). Kong et al. (Kong et al., 2016) reported that platycodin D treatment in human breast MDA-MB-231 cells induces cell cycle arrest at G0/G1 phase through inhibition of MDM2. Zhou et al. (Zhou et al., 2015) also suggested that platycodin D stimulates the expression of the transcription factor, FOXO3a, which triggers apoptosis through up-regulation of TRAIL (Modur et al., 2002). Here, our data demonstrated that up-regulation of PUMA expression was critical for platycodin D-induced apoptosis (Figures 2, 4) since knockdown of PUMA partially abolished the platycodin D-increased cleaved caspase 3 and apoptosis (Figure 4). In contrast to induction of apoptosis, recent studies also suggested that platycodin D can protect oxidative stress damage in non-tumorigenic cells. For example, cardiomyocyte H9c2 cells treated with platycodin D reduce hypoxia/reoxygenation-induced oxidative stress (Wang et al., 2018). Platycodin D also decreases oxidative stress induced by cisplatin treatment in HEK293 cells (Hu et al., 2021). Taken together, platycodin D seems to protect normal cells from oxidative stress damage while it induces cell death in cancer cells, suggesting that platycodin D is a promising agent for cancer therapeutics.

JNKs belong to the mitogen-activated protein kinase superfamily, which consists of JNK1, JNK2, and JNK3. The JNK1 and JNK2 are expressed in all tissues while JNK3 is limited to the brain, heart, and testes (Davis, 2000). Both JNK1 and JNK2 have been implicated in the induction of apoptosis in response to different cellular stimuli. For example, JNK1 mediates TNF-α and UV-induced apoptosis in mouse embryonic fibroblasts (Liu et al., 2004), and JNK2 is involved in pheochromocytoma PC12 cell death in response to 6-hydroxydopamine (Eminel et al., 2004). In consistence with this notion, we found that inhibition of JNK by SP600125 attenuated platycodin D-induced apoptosis (Figure 5). Furthermore, knockdown of JNK1 but not JNK2 partially blocked platycodin D-induced PUMA expression and cleaved caspase 3 level, indicating that JNK1 is essential for platycodin D-induced apoptosis (Figure 5). Interestingly, our data also suggested that only phosphorylation of c-Jun at Ser63 but not Ser73 was induced phosphorylation upon platycodin D treatment, and the level of phospho-c-Jun (Ser63) was dramatically decreased by JNK1 knockdown, suggesting that phosphorylation of c-Jun at Ser 63 activated AP-1 to promote PUMA transcription in response to platycodin D treatment. The mechanism for platycodin D only inducing c-Jun phosphorylation at Ser63 but not Ser73 needs to be further investigated.

In addition to promoting apoptosis, JNK/AP-1 also plays a critical role in promoting cell proliferation and tumorigenesis. Epoxyeicosatrienoic acid activates JNK/AP-1 pathway to promote cell proliferation and prevent apoptosis in pulmonary artery endothelial cells (Ma et al., 2012). Besides, activation of the JNK/AP-1 axis by over-expression of Jun or Fos proteins enhances invasion and metastasis (Eferl and Wagner, 2003). How does JNK control cell proliferation or apoptosis? Ventura et al. (Ventura et al., 2006) showed that short-term (<1 h) activation of JNK by TNF-α leads to survival, whereas the prolonged activation of JNK results in apoptosis. These observations suggested that the duration of JNK activation may control the cell fate, i.e., survival or apoptosis. It is also possible that regulation of apoptosis or proliferation by JNK/AP-1 is in a cell- or tissue-specific manner. However, the detailed mechanism needs to be further explored.

## 5 Conclusion

In conclusion, activation of the JNK1/AP-1/PUMA axis by platycodin D is essential for apoptosis induction in suppression of NSCLC growth, providing a new mechanism of how platycodin D suppresses NSCLC.

## Data Availability

The raw data supporting the conclusions of this article will be made available by the authors, without undue reservation.

## Abbreviations

NSCLC, Non-small lung cancer cells; PUMA, p53 upregulated modulator of apoptosis; Bcl-2, B cell lymphoma 2; Bax, Bcl-2 associated X protein; BH3, Bcl-2 homology 3; JNK, Jun N-terminal kinase; OCR, Oxygen consumption rate; SRC, Spare respiratory capacity; CHIP, Chromatin immunoprecipitation
