# Supplementary material for: Platycodin D induces apoptosis through JNK1/AP-1/PUMA pathway in non-small cell lung cancer cells: A new mechanism for an old compound
Source: Front Pharmacol. 2022 Nov 22;13:1045375. doi: 10.3389/fphar.2022.1045375 (PMC9723146; doi:10.3389/fphar.2022.1045375)
Supplement: Supplementary file 1 [file DataSheet1.docx]

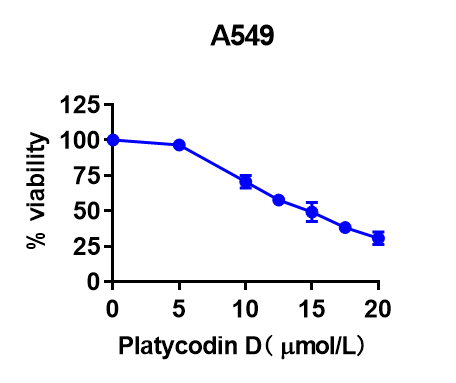


**Supplementary** **Fig. 1.** Platycodin D inhibits A549 cell viability. Cells were treated with platycodin D (0 to 20 μmol/L) for 48 h and cell viability was determined by CellTiter-Glo® Luminescent Cell Viability Assay kit. The bioluminescent signal at 0 μmol/L is designated as 100%. Results are expressed as mean ± SD (n=4).


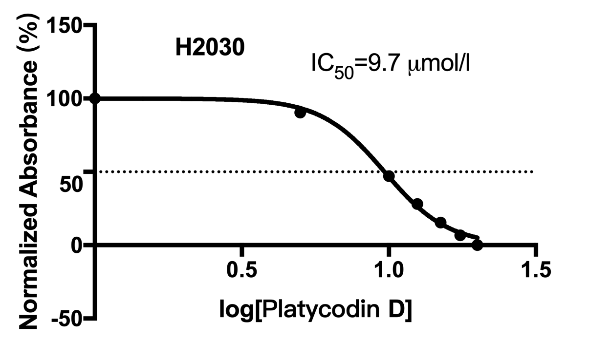

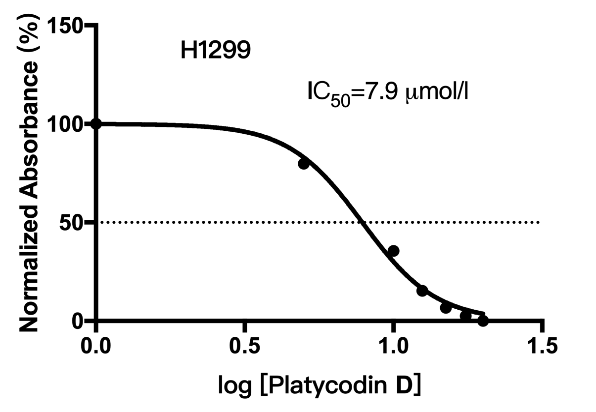

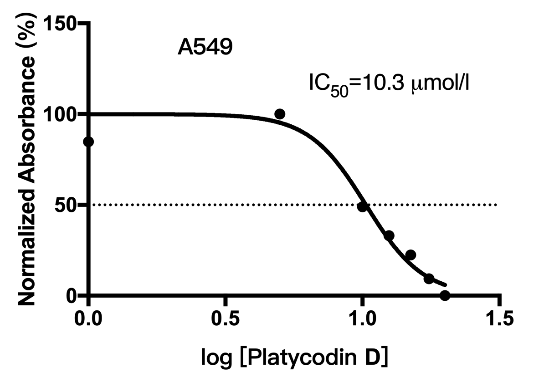


**Supplementary Fig. 2.** IC_50_ values of platycodin D in H1299, H2030, and A549 cells. The IC_50_ values were calculated from the results of **Fig. 1A** using GraphPad Prism 9.

**A**

**GAPDH**

**H2030**

**cl-caspase-3**


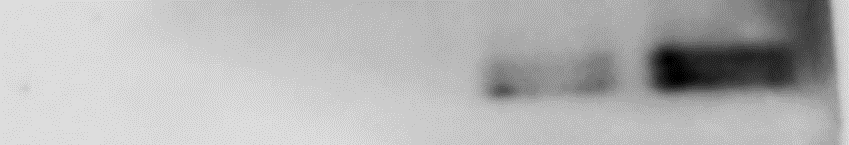

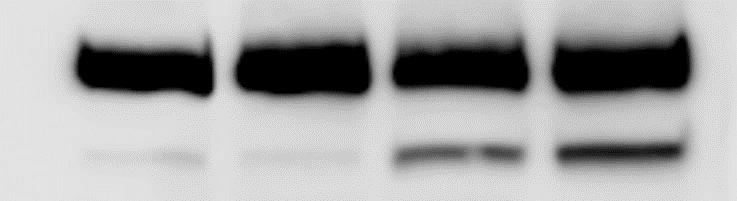

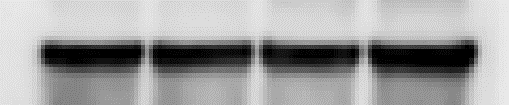


**PD (μmol/L)**

**PARP**

**cl-PARP**

**0 5 10 15**

**19 kDa, 17 kDa**

**116 kDa**

**89 kDa**

**37 kDa**

**B**


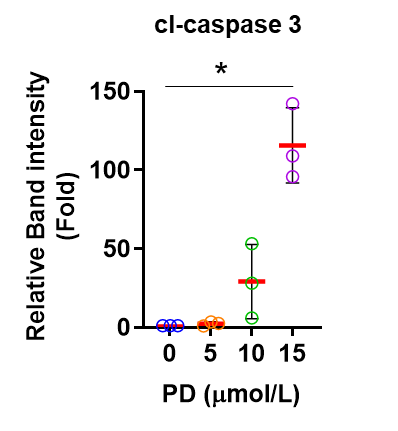

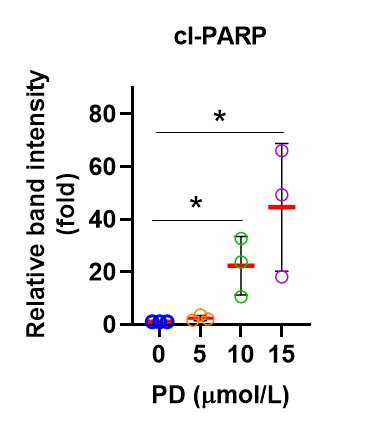


**Supplementary Fig. 3.** Platycodin D treatment increases the level of cleaved PARP and cleaved caspase 3 in H2030 cells. (A) H2030 cells were treated with 0 to 15 μmol/L of platycodin D for 48 h and subsequently used for Western blot analyses. cl-PARP: cleaved PARP; cl-Caspase 3: cleaved caspase 3. (B) Densitometric quantification of cleaved PARP and cleaved caspase 3 levels in (A). The ratio of cleaved PARP/GAPDH or cleaved caspase 3/GAPDH in vehicle (0 μmol/L) treated cells is designated as 1. Data were analyzed using one sample *t* test (mean ±SD, n=3, *P < 0.05; ***P < 0.001).


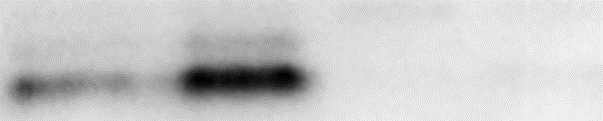


**23kDa**

**19kDa, 17kDa**

**37kDa**

**PUMA**

**cl-caspase 3**

**GAPDH**

**siPUMA**

**scrambled**

**PD**

**siPUMA**

**scrambled**

**Vehicle**


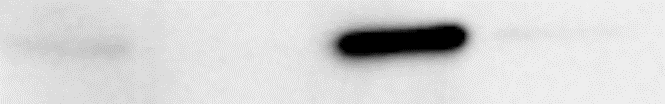

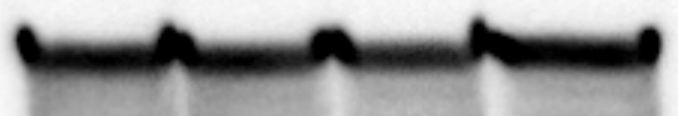


**Supplementary Fig. 4.**  Knockdown of PUMA suppresses platycodin D-induced cleavage of caspase 3 in H2030 cells. Cells were treated with 15 μmol/L of Platycodin D for 24 h and subsequently used for Western blot analyses. cl-Caspase 3: cleaved caspase 3.


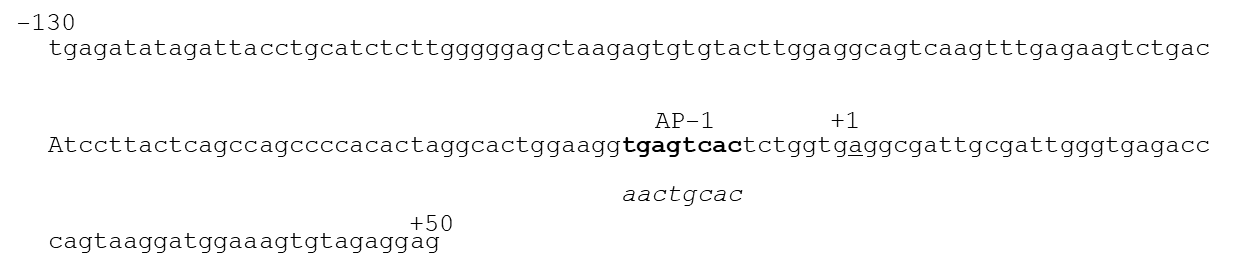


**Supplementary Fig. 5.** The nucleotide sequence of PUMA promoter from -130 bp to +50 bp. The AP-1 site is bolded and the mutated AP-1 sequence is italic. The transcription start site is underlined.


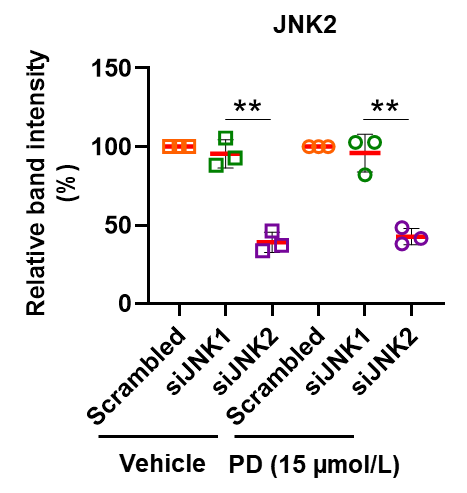

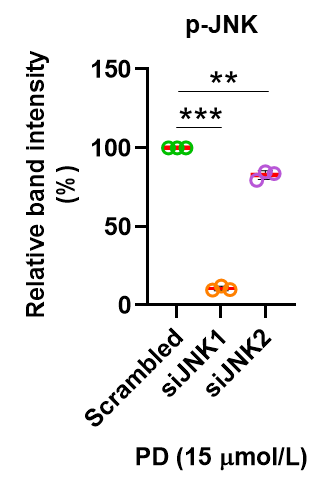

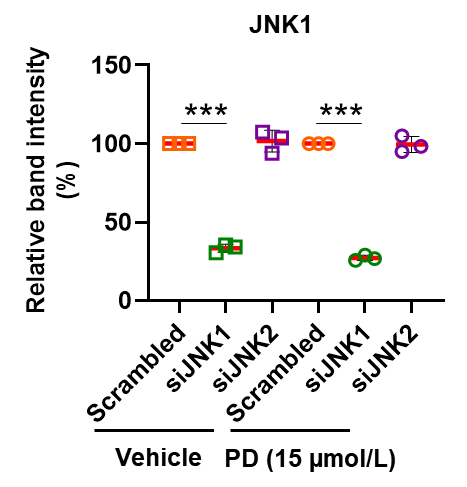


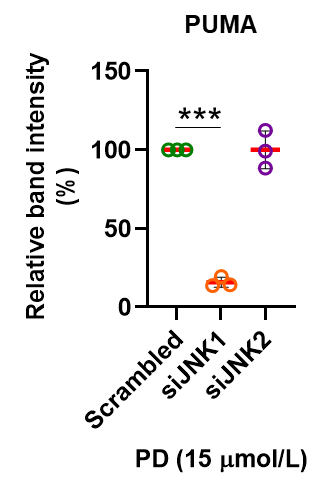

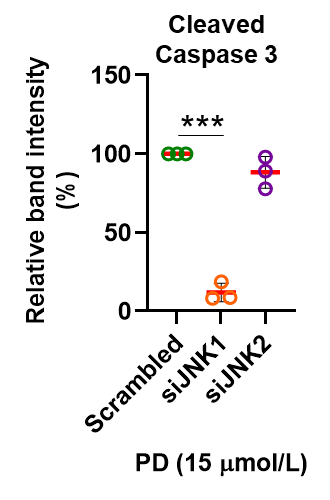

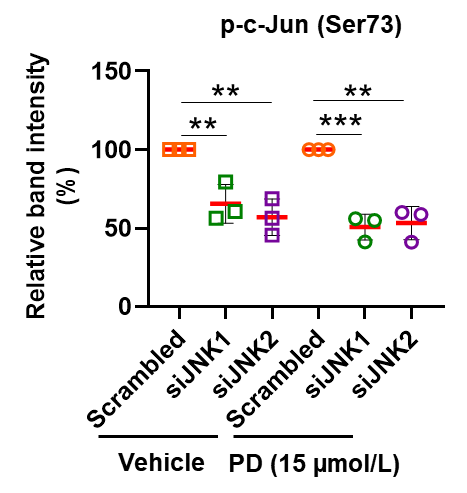


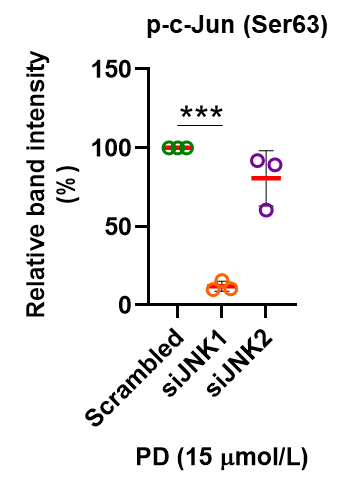


**Supplementary Fig. 6.** Densitometric quantification of the levels of JNK1, JNK2, phospho-JNK, phospho-c-Jun (Ser63), phospho-c-Jun (Ser73), PUMA, and cleaved caspase 3 in Fig. 5f. The ratio of target protein/GAPDH in scrambled siRNA transfected cells is designated as 100. Data were analyzed using one sample *t* test (mean ±SD, n=3, **P < 0.01; ***P < 0.001).
